# Ultrafast Laplace NMR to study metal–ligand interactions in reversible polarisation transfer from parahydrogen†

**DOI:** 10.1039/d1cp02383g

**Published:** 2021-07-22

**Authors:** Ben. J. Tickner, Vladimir V. Zhivonitko, Ville-Veikko Telkki

**Affiliations:** NMR Research Unit, Faculty of Science, University of Oulu 90014 Finland vladimir.zhivonitko@oulu.fi ville-veikko.telkki@oulu.fi

## Abstract

Laplace Nuclear Magnetic Resonance (NMR) can determine relaxation parameters and diffusion constants, giving valuable information about molecular structure and dynamics. Information about relaxation times (*T*_1_ and *T*_2_) and the self-diffusion coefficient (*D*) can be extracted from exponentially decaying NMR signals by performing a Laplace transform, which is a different approach to traditional NMR involving Fourier transform of a free induction decay. Ultrafast Laplace NMR uses spatial encoding to collect the entire data set in just a single scan which provides orders of magnitude time savings. In this work we use ultrafast Laplace NMR *D*–*T*_2_ correlation sequences to measure key relaxation (*T*_2_) and diffusion (*D*) parameters of methanolic solutions containing pyridine. For the first time we combine this technique with the hyperpolarisation technique Signal Amplification By Reversible Exchange (SABRE), which employs an iridium catalyst to reversibly transfer polarisation from parahydrogen, to boost the ^1^H NMR signals of pyridine by up to 300-fold. We demonstrate use of ultrafast Laplace NMR to monitor changes in pyridine *T*_2_ and *D* associated with ligation to the iridium SABRE catalyst and kinetic isotope exchange reactions. The combined 1440-fold reduction in experiment time and 300-fold ^1^H NMR signal enhancement allow the determination of pyridine *D* coefficients and *T*_2_ values at 25 mM concentrations in just 3 seconds using SABRE hyperpolarised ultrafast Laplace NMR.

## Abstract 2

Ultrafast Laplace NMR can monitor changes in ligand dynamics due to metal ligation and isotope exchange. A 300-fold sensitivity boost from SABRE hyperpolarisation can provide a 1440-fold time saving in determination of *D* and *T*_2_.

## Graphical abstract

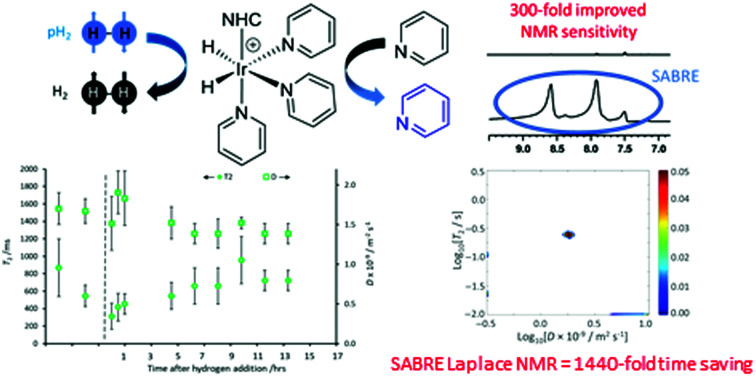

## Introduction

**Fig. 1:**
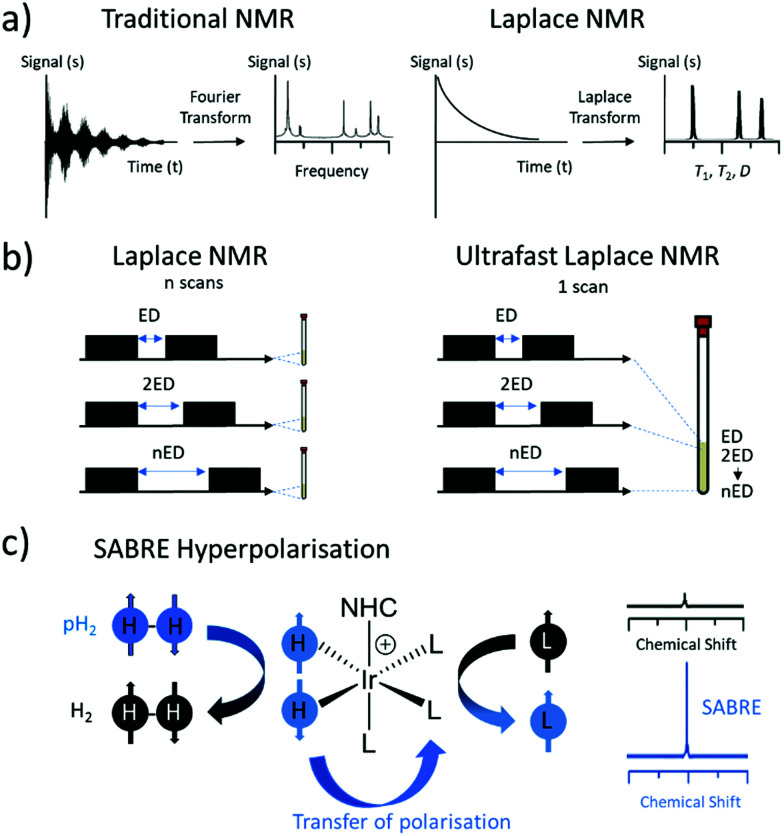
Summary of Laplace NMR, spatial encoding approach for ultrafast experiments and SABRE hyperpolarisation. (a) Traditional NMR (left) involves recording a free induction decay which is then Fourier transformed to generate frequency information. In Laplace NMR (right), exponentially decaying signals are recorded which undergo Laplace inversion to generate distributions of *T*_1_, *T*_2_ or *D*. (b) Conventional Laplace NMR involves recording many repetitions with variable evolution delays (ED) between preparation and mixing blocks (left). In the case of ultrafast LNMR, spatial encoding is used to record the entire dataset in just a single scan (right). (c) SABRE hyperpolarisation involves transfer of parahydrogen-derived magnetisation to a target ligand (L) *via* reversible interactions with an iridium catalyst (left) to boost NMR signals (right).

Magnetic Resonance (MR) is one of the most widely used techniques for the characterisation of molecules and materials. Measurement of self-diffusion coefficients, *D*, or nuclear spin relaxation times, *T*_1_ and *T*_2_, can give information about molecular structure, dynamics and mobility in a non-invasive manner.^1^ A set of NMR methods known as Laplace NMR (LNMR) can be used to measure a range of parameters including *D*, *T*_1_ (time taken to establish equilibrium longitudinal magnetisation) and *T*_2_ (time taken to dephase transverse magnetisation).^2^ This is achieved by measuring exponentially decaying signals whose time dependence is reliant on relaxation or diffusion. The distribution of relaxation times or diffusion coefficients can be extracted from LNMR experiments by performing an inverse Laplace transform of the observed decaying signals.^3,4^ This is different to traditional NMR experiments which typically measure a free induction decay (FID) following radiofrequency excitation(s) to give chemical shift and frequency information upon Fourier Transform (Fig. 1a).^5^

Multidimensional LNMR methods allow correlations to be made between relaxation times and diffusion constants. For example, *T*_1_–*T*_2_ correlation,^6^*D*–*T*_2_ correlation,^7^*T*_2_–*T*_2_ exchange^8^ and *D*–*D* exchange^9^ have been developed and used to gather information about molecular structure and dynamics. Even 3D, and higher dimensional,^10^ LNMR experiments have been reported that show correlations between *T*_1_, *T*_2_ and *D*.^11^ However, multidimensional LNMR experiments must be repeated 10–1000 times to probe the indirect dimension and are therefore limited by long acquisition times which can be between several hours to a few days.

One approach to address these issues is the development of layered spatially encoded NMR which allows an entire dataset to be collected in just a single scan (Fig. 1b).^12,13^ For example, *T*_1_^14^ and *D*^15,16^ experiments can be collected using this approach in 1 scan. This has been used to develop ultrafast Laplace NMR experiments^17–19^ which reduce the experiment time by one to four orders of magnitude compared to traditional approaches.^2^ Consequently, ultrafast LNMR has been used to study molecular dynamics in a range of chemical systems including porous materials,^17,20^ cellular metabolites,^21^ and surfactant aggregates.^22^ Nevertheless, these measurements can be limited by the low sensitivity of MR which is derived from perturbations of very small population differences across closely spaced nuclear spin energy levels. In fact, an astonishingly low number of nuclear spins (only 1 of every 32 000 ^1^H spins at 9.4 T) contribute to the MR signals detected using ultrafast LNMR and more traditional NMR methods. Low signal intensity often makes it challenging to differentiate between different components in LNMR experiments that contain small differences between their relaxation times or diffusion constants. Greater resolution between different components is generally achieved by higher signal to noise ratios.^17^

To improve the sensitivity, hyperpolarisation (HP) techniques can be used to create non-Boltzmann population distributions across nuclear spin energy levels.^23^ As a result, HP MR signals can be up to five orders of magnitude larger than those derived from Boltzmann populated systems. There are a wide range of hyperpolarisation techniques, but in this work we focus on the use of signal amplification by reversible exchange (SABRE) which is able to generate enhanced magnetisation in a low-cost and refreshable manner (Fig. 1c).^24^ SABRE achieves this by using parahydrogen (pH_2_), a spin isomer of hydrogen gas, as its source of nuclear spin order. Hydrogen gas can easily be enriched in its para isomer by cooling to low temperature (here 92% enrichment at 37 K) in the presence of a spin exchange catalyst (Fe_2_O_3_). Subsequent heating and removal of the spin exchange catalyst yields enriched pH_2_ suitable for use in HP studies. SABRE relies on a reversible oxidative addition reaction involving an iridium centre to break the symmetry of pH_2_.^24^ This is necessary to unlock the latent magnetism of pH_2_, which can then be transferred to ^1^H or heteronuclear sites of ligated molecules within the active catalyst.^24^ This transfer occurs through a temporary *J*-coupled network^25–28^ formed between pH_2_-derived hydride ligands and target sites at low magnetic field (mT or μT for transfer to ^1^H^24,29,30^ or X-nuclei^31–34^ respectively). Reversible dissociation yields hyperpolarised ligand free in solution. As SABRE is catalytic, it can produce hyperpolarised molecules in a cheap and continuous process, which is an advantage compared to alternative HP techniques that are typically batch processes.^23,35^

Single scan ultrafast LNMR is ideally suited for detection of hyperpolarised substances as the dataset can be recorded rapidly before relaxation of enhanced nuclear spin polarisation back to their Boltzmann-derived states.^17,20,21,36^ In this work we use SABRE HP to provide enhanced ^1^H NMR signals for the substrate pyridine, which we then use to collect single scan ultrafast LNMR experiments based on *D*–*T*_2_ correlation. We have selected pyridine as it was one of the first molecules to be hyperpolarised using the SABRE technique in 2009^24^ and since then has become one of the most studied substrates for SABRE HP.^29,37–40^ Our aim is to use SABRE hyperpolarised ultrafast LNMR to record diffusion and relaxation parameters with orders of magnitude time savings and sensitivity improvements compared to use of standard NMR, or even thermally polarised ultrafast LNMR.

## Experimental and methods

### *D*–*T*_2_ pulse sequences

**Fig. 2:**
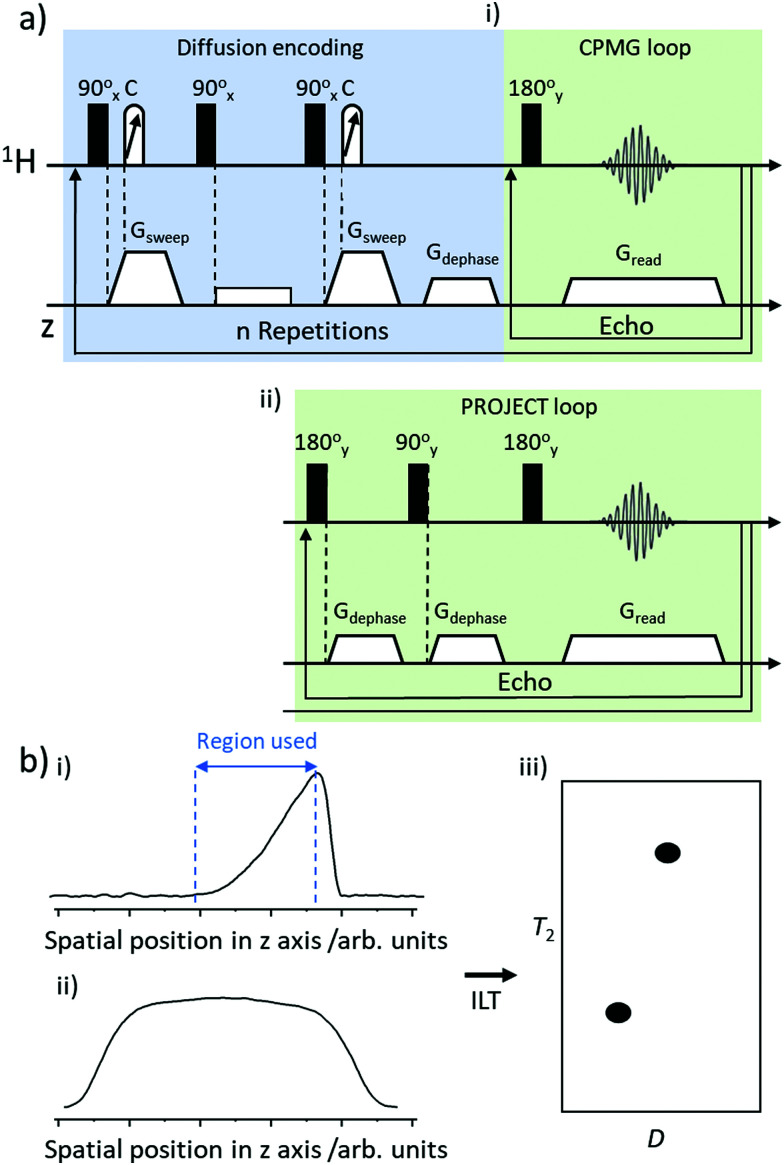
Summary of ultrafast Laplace NMR pulse sequence and method for extraction of *D* and *T*_2_ (a) pulse sequence for *D*–*T*_2_ correlation experiments which consist of a diffusion encoding block followed by either a (i) CPMG loop or (ii) PROJECT loop. (b) Generic depiction of (i) spatially encoded *D*–*T*_2_ detection which are referenced to (ii) a coil sensitivity profile to generate (iii) *D*–*T*_2_ correlation spectra using an inverse Laplace Transform (ILT).

In this work we use *D*–*T*_2_ correlation experiments to measure *D* and *T*_2_ for thermally polarised and SABRE-hyperpolarised pyridine. Pulse sequences for these *D*–*T*_2_ correlation experiments have been reported elsewhere^17,21,36^ and are summarised in Fig. 2a. Briefly, they contain a PGSTE (pulsed field gradient stimulated echo) to achieve diffusion encoding which is followed by either a CPMG (Carr–Purcell–Meiboom–Gill)^41^ or PROJECT (Periodic Refocusing of *J* Evolution by Coherence Transfer)^42^ loop to encode *T*_2_ relaxation.^17^

The ultrafast *D*–*T*_2_ pulse sequence was set with a spin echo time of 15 ms and a big delta value of 50 ms. For sequences containing a PROJECT loop the corresponding double spin echo time was 30 ms. The chirp pulses used contained a bandwidth of 150 000 Hz and a length of 2 ms. The length of the hard π/2 pulse was 8.6 μs. The number of echoes was 64, and the number of scans was typically 32, with a repetition time of 20 s (taking a total of 11 min). Each echo was acquired with 256 complex points. For SABRE HP LNMR measurements, only a single scan was used taking just 3 seconds. The acquisition was followed by a Fourier transform in the spatial frequency dimension and removal of data outside the spatial encoding region.

Spatially encoded detection along the *z*-direction can be influenced by the excitation–detection sensitivity profile of the radiofrequency coil. This can be negated by recording a reference 1D experiment of the sample along the *z*-axis with the same parameters as the CPMG loop of the ultrafast *D*–*T*_2_ sequence. Data were processed using a custom Matlab script that selects the region of spatially encoded exponential signal decay, corrects to the coil sensitivity profile and then performs a 2D inverse Laplace transform (summarised in Fig. 2b). The algorithm based on adaptive truncation of matrix decompositions was used for Laplace inversion.^43^ The value of the parameter (*α*) scaling the weight of the Tikhonov regularisation was adjusted in a standard way by running the *α* loop. Errors in *D* and *T*_2_ were estimated based on the width of the peak in 2D *D*–*T*_2_ correlation spectra.

All NMR measurements were carried out on a 400 MHz Bruker Avance III spectrometer using solutions at room temperature (298 K). ^1^H (400 MHz) NMR spectra were recorded with an internal deuterium lock. Chemical shifts are quoted as parts per million and referenced to the solvent C*H*D_2_OD signal at *δ* 3.34 ppm. 1D DOSY experiments used a big delta value of 200 ms and a little delta of 1000 μs. The number of echoes was 32, and the number of scans was typically 4, with a repetition time of 15 s (taking a total of 36 min). For *T*_2_ measurements recorded using 1D CPMG sequences the echo time was 40 ms with 12 echoes and a repetition time of 20 s and 8 scans (taking a total of 36 min). Therefore, the thermally polarised (11 min) and SABRE hyperpolarised (3 seconds) ultrafast LNMR experiments used in this work provide a 6.5-fold and 1440-fold time saving respectively compared to measuring *D* and *T*_2_ with conventional NMR experiments (36 min each).

### Synthetic procedures

All commercial compounds were purchased from Sigma-Aldrich and used as supplied unless otherwise stated. [IrCl(COD)(IMes)] (where IMes = 1,3-bis(2,4,6-trimethyl-phenyl)imidazole-2-ylidene and COD = *cis*,*cis*-1,5-cyclooctadiene) was synthesised according to literature procedures.^44^ SABRE samples were prepared containing 2 mg [IrCl(COD)(IMes)] precatalyst and the indicated amount of pyridine in 0.6 mL of methanol-*d*_4_ in a 5 mm NMR tube that was fitted with a quick pressure valve. The resulting solutions were degassed by three freeze–pump–thaw cycles before the addition of 3 bar pH_2_. Hydrogen gas was produced using a desktop hydrogen generator (F-DGSi, Evry, France). This was used to make parahydrogen (pH_2_) using a BPHG 90 parahydrogen generator (Bruker). Samples were left to activate for a period of several hours (usually around 16 hours overnight). Activation is usually indicated by a change in colour from orange to pale orange and the formation of a peak in ^1^H NMR spectra at *δ* = −22.74 ppm corresponding to [Ir(H)_2_(IMes)(pyridine)_3_]Cl.^24^

### SABRE experiments

The shake and drop method was employed for recording hyperpolarised NMR spectra. This involves filling NMR tubes with fresh pH_2_ (3 bar) before shaking them vigorously for 10 seconds in a 6.5 mT (65 G) magnetic field.^24^ These fields are provided by an electromagnetic coil powered by a Blanko PS-3005 0–30 V 0–5 A switching power supply. After shaking, the sample is rapidly inserted into the spectrometer before data collection. More details of this apparatus and procedure are provided in the ESI† (Fig. S11). ^1^H NMR signal enhancements were calculated by dividing the hyperpolarised signal integral intensity by their corresponding integrals in a thermally polarised spectrum. It is essential that both spectra are recorded and processed using the same spectral acquisition parameters. Multiple shake and drop measurements were undertaken and average signal enhancement values quoted.

## Results and discussion

### Using ultrafast Laplace NMR to measure *D* and *T*_2_ of concentrated thermally polarised pyridine

**Fig. 3:**
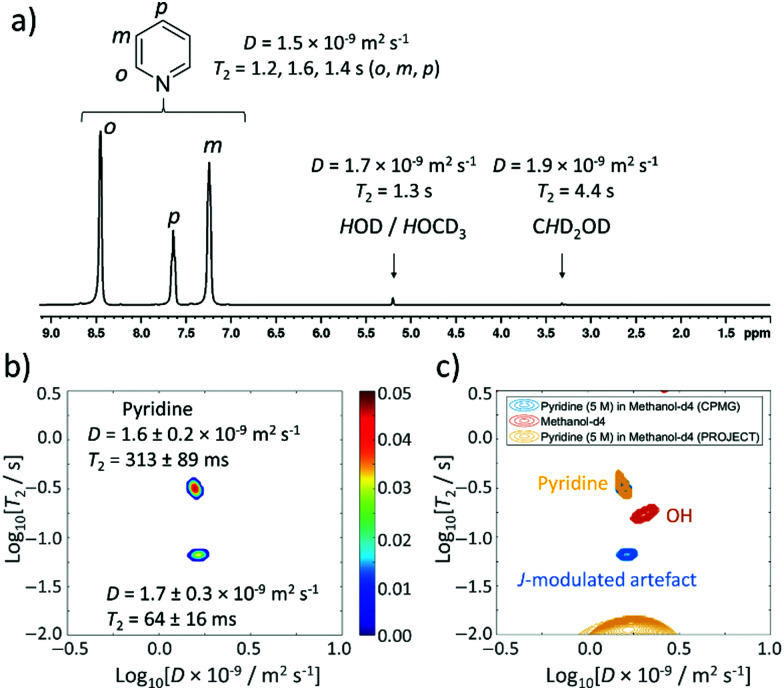
Thermally polarised ultrafast Laplace NMR of 5 M pyridine provides a 6.5-fold time saving compared to conventional NMR. (a) ^1^H NMR spectrum with *D* and *T*_2_ values determined from 1D DOSY and CPMG experiments (36 min each), respectively. (b) *D*–*T*_2_ correlation (with CPMG loop, 11 min) of pyridine (5 M) in methanol-*d*_4_. (c) *D*–*T*_2_ correlation plot from (b) (blue) overlaid with one recorded using a PROJECT loop (yellow). A reference sample containing only methanol-*d*_4_ (0.6 mL) recorded using a CPMG loop is also shown (orange). Additional spectra are presented in the ESI,† Fig. S1.

This study begins with the measurement of relaxation times and diffusion coefficients of pyridine, which is perhaps the most commonly studied substrate for the SABRE hyperpolarisation technique.^24,29,37–40^^1^H NMR spectroscopy of this solution yields signals for the *ortho*, *meta* and *para* pyridine sites at *δ* 8.50, 7.30 and 7.68 ppm respectively. Additional signals corresponding to residual methanol-*d*_4_ are also present at *δ* 5.25 (*H*OCD_3_) and 3.34 ppm (C*H*D_2_OD) (Fig. 3a). The peak at *δ* 5.25 ppm is also expected to contain a contribution from the presence of any contaminant water in these mixtures which arises from the use of methanol-*d*_4_ solvent that has not been vigorously dried and is in exchange with the methanol solvent.^45^ The integral intensities of these signals are present in a pyridine : *H*O : C*H*D_2_OD ratio of 275 : 3 : 1. To start with, reference 1D measurements of *D* and *T*_2_ for these molecules in this mixture were recorded using DOSY (diffusion ordered spectroscopy) and a standard CPMG sequence (these values are shown in Fig. 3a).

This concentrated solution of pyridine (5 M) in methanol-*d*_4_ (0.6 mL) was also used for determination of *D* and *T*_2_ using the ultrafast LNMR pulse sequences shown in Fig. 2a. In the case of *D*–*T*_2_ sequences recorded using a CPMG loop, a signal profile is recorded which upon Laplace transformation yields two signals (Fig. 3b). The dominant of these signals has a *D* coefficient (*D* = 1.6 ± 0.2 × 10^−9^ m^2^ s^−1^) that is comparable to that of pyridine confirmed by the reference measurements (*D* = 1.5 × 10^−9^ m^2^ s^−1^). We therefore assign this dominant peak as arising from pyridine. The *T*_2_ value of this signal determined using UF LNMR sequences (313 ± 89 ms) is significantly shorter than those of pyridine measured from reference 1D measurements (1123, 1324, 931 ms for the *ortho*, *meta* and *para* sites respectively). Similar compression of real *T*_2_ values to give shorter apparent *T*_2_ values using these *D*–*T*_2_ UF LNMR sequences has been reported previously and is an effect of diffusion-induced signal attenuation during the CPMG loop due to detection gradients.^17,20^ The second peak appearing in these UF LNMR *D*–*T*_2_ correlation plots contains a similar diffusion constant (*D* = 1.7 ± 0.3 × 10^−9^ m^2^ s^−1^) and a shorter *T*_2_ (64 ± 16 ms). Such a signal could arise from either the methanol/water component, or it could be an artefact caused by pyridine homonuclear *J* coupling.

In order to gain more insight and confirm these assignments, *D*–*T*_2_ UF LNMR sequences were used containing a PROJECT loop (Fig. 2aii), which have been reported to remove the effect of *J* modulation compared to CPMG loops.^17,42^ Use of these sequences resulted in the observation of only one peak in *D*–*T*_2_ correlation spectra with *D* and *T*_2_ values of 1.5 ± 0.4 × 10^−9^ m^2^ s^−1^ and 343 ± 40 ms respectively (Fig. 3c). This suggests that the appearance of a second minor peak in *D*–*T*_2_ UF LNMR sequences containing a CPMG loop is likely due to *J*-modulation effects which are removed by inclusion of a PROJECT loop in the *D*–*T*_2_ sequence.

It is worth noting that when a solution of methanol-*d*_4_ solvent (0.6 mL), that does not contain pyridine, is examined using *D*–*T*_2_ UF LNMR (with a CPMG loop), a single signal is observed with *D* and *T*_2_ values of 2.0 ± 0.4 × 10^−9^ m^2^ s^−1^ and 174 ± 40 ms respectively (Fig. 3c and ESI,† Fig. S1b). This confirms that the background solvent matrix can give rise to additional signals in these experiments. These solvent signals do not overlap with those observed in the pyridine-containing sample (Fig. 3c), which confirms that the additional signal that appears when pyridine (5 M) is used is related to *J*-modulation artefacts. The reference *T*_2_ values reported here are collected using 1D CPMG data that can also exhibit the effect of *J*-modulation (see ESI,† Section S3).

### Using ultrafast Laplace NMR to measure *D* and *T*_2_ of dilute solutions of thermally polarised pyridine

**Fig. 4:**
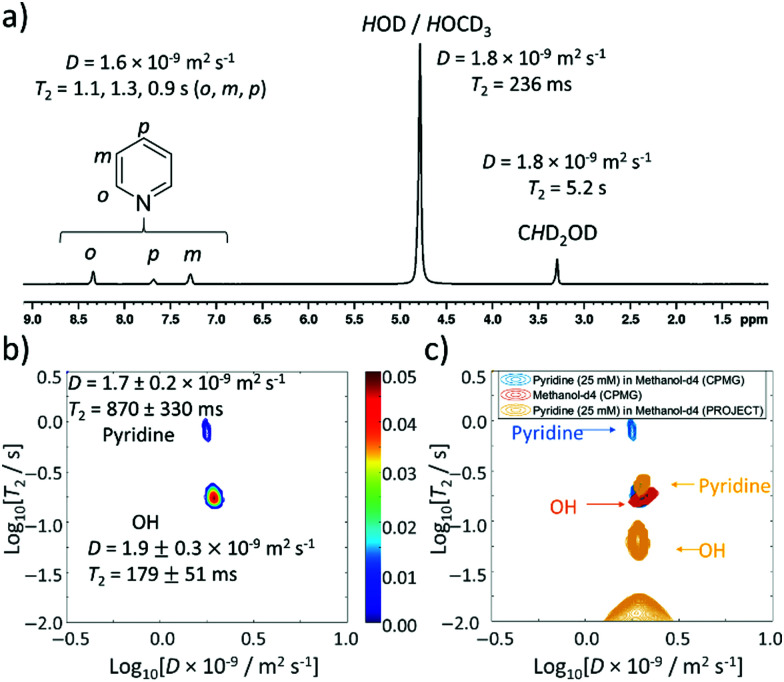
Thermally polarised ultrafast Laplace NMR of 25 mM pyridine provides a 6.5-fold time saving compared to conventional NMR. (a) ^1^H NMR spectrum with *D* and *T*_2_ values determined from 1D DOSY and CPMG experiments (36 min each), respectively (b) *D*–*T*_2_ correlation (with CPMG loop, 11 min) of pyridine (25 mM) in methanol-*d*_4_ (c) *D*–*T*_2_ correlation plot from (b) (blue) overlaid with that recorded using a PROJECT loop (yellow). A reference sample containing only methanol-*d*_4_ (0.6 mL) recorded using a CPMG loop is also shown (orange). Additional spectra are presented in the ESI,† Fig. S2.

In SABRE experiments, the ratio between pyridine and the iridium polarisation transfer catalyst is carefully controlled as this can affect the achieved NMR signal enhancement *via* ligand exchange and relaxation effects. Typically, substrate : metal ratios of between 4 : 1 and 10 : 1 are used to achieve most optimal SABRE hyperpolarisation.^24,29,37^ Iridium concentrations of *ca.* 5 mM are commonly used as this gives an appropriate metal : H_2_ ratio for parahydrogen exchange that must occur for magnetisation within the active SABRE catalyst to be refreshed.^46,47^ As a consequence of these considerations, substrate concentrations on the order of tens of mM are typically used in conjunction with SABRE experiments. Therefore, a more dilute sample of pyridine (25 mM) in methanol-*d*_4_ was prepared. Now, ^1^H NMR spectra show a pyridine : *H*O : C*H*D_2_OD signal intensity ratio of 2 : 14 : 1. Reference measurements of this mixture show similar values of *D* (1.6 × 10^−9^ m^2^ s^−1^) and *T*_2_ (1.1, 1.3 and 0.9 s for *ortho*, *meta*, and *para* sites respectively) for pyridine (Fig. 4a) compared to the 5 M pyridine sample discussed earlier. Interestingly, the *T*_2_ value of the O*H* signal at *δ* 5.25 ppm is compressed from 1.3 s (5 M pyridine) to 236 ms (25 mM pyridine).

When *D*–*T*_2_ sequences are employed, two signals are again observed (Fig. 4b). For *D*–*T*_2_ sequences recorded using a CPMG loop, a dominant signal is now visible with a *D* coefficient (*D* = 1.9 ± 0.3 × 10^−9^ m^2^ s^−1^) and *T*_2_ value (179 ± 51 ms) that closely match those measured for both the *H*OD/*H*OCD_3_ signal by 1D measurements of this sample (*D* = 1.8 × 10^−9^ m^2^ s^−1^ and *T*_2_ = 236 ms) and UF LNMR measurements on the reference sample of methanol-*d*_4_ discussed earlier (*D* = 2.0 ± 0.4 × 10^−9^ m^2^ s^−1^ and *T*_2_ = 174 ± 40 ms) (Fig. 4c). We therefore assign this major signal to the background solvent and note that its dominance in solutions of dilute pyridine is a consequence of the fact that its ratio relative to pyridine is now much higher. The second peak appearing in these UF LNMR *D*–*T*_2_ correlation plots, which is not present with significant intensity, contains a diffusion constant (*D* = 1.7 ± 0.2 × 10^−9^ m^2^ s^−1^) and *T*_2_ value (870 ± 330 ms) consistent with those of pyridine. In these dilute samples we no longer notice the appearance of *J*-modulated artefacts (likely due to low pyridine signal intensity) and confirm from the use of *D*–*T*_2_ sequences containing a PROJECT loop that two signals are still present, albeit with compressed *T*_2_ values relative to the CPMG-containing analogue (Fig. 4c). This may be related to the effect of greater diffusion during the longer double spin echo.

### Formation of SABRE catalysts examined using thermally polarised ultrafast Laplace NMR

**Fig. 5:**
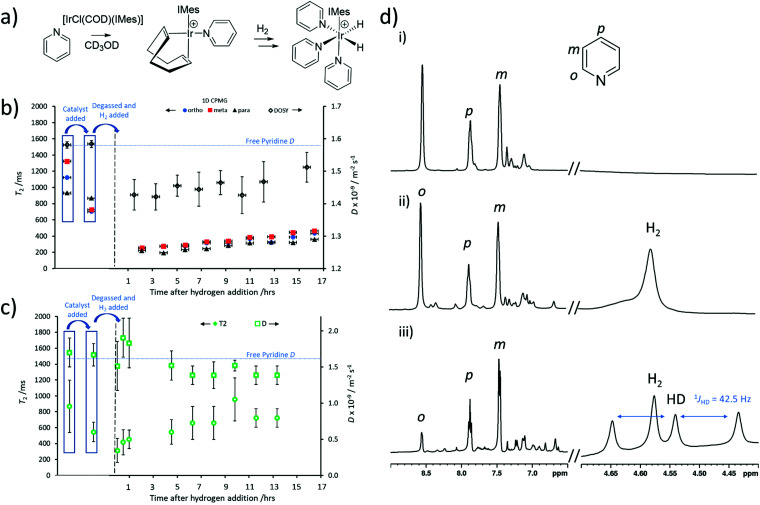
Thermally polarised reaction monitoring of pyridine reactivity associated with SABRE. (a) Formation of the active SABRE catalyst. Changes in *T*_2_ and *D* measured from (b) 1D CPMG and DOSY experiments respectively (36 min each) and (c) ultrafast Laplace NMR (11 min) following addition of 3 bar H_2_ to a solution of [IrCl(COD)(IMes)] (5 mM) and pyridine (5 equiv.) in methanol-*d*_4_ (0.6 mL). In (b) *D* for the three pyridine sites are averaged and the error bar reflects the spread in these values. In (c) error bars are determined from the width of the peak in *D*–*T*_2_ correlation spectra. Horizontal error bars in (b) reflect the measurement time and are 6.5 times longer than those in (c) for which error bars are narrower than the marker width. (d) ^1^H NMR spectra of [IrCl(COD)(IMes)] (5 mM) and pyridine (5 equiv.) in methanol-*d*_4_ (0.6 mL) (i) prior to H_2_ addition (ii) *ca.* 10 min and (iii) *ca.* 18 hours after H_2_ (3 bar) addition.

SABRE measurements require formation of an active polarisation transfer catalyst, which for N-heterocyclic substrates such as pyridine is typically of the form [Ir(H)_2_(IMes)(pyridine)_3_]Cl.^24^ These species are formed *in situ* from a [IrCl(COD)(IMes)] precatalyst which first reacts with substrate (in this case pyridine) to form [Ir(COD)(IMes)(pyridine)]Cl.^48^ Then, after H_2_ addition, [Ir(H)_2_(COD)(IMes)(pyridine)]Cl is initially formed which eventually converts into [Ir(H)_2_(IMes)(pyridine)_3_]Cl (Fig. 5a).^48^ This activation process is usually monitored using ^1^H NMR spectroscopy, although changes in substrate *T*_1_ have been used to track these reactions.^49^ For example, binding of substrate to the metal centre is known to cause compression of substrate *T*_1_,^50,51^ and therefore can impart information on the progress of this reaction.^49^ It might be expected that substrate *T*_2_ may change in a similar fashion. Changes in effective molecular size may also result in changes in *D.*

Therefore, a sample of [IrCl(COD)(IMes)] (5 mM) and pyridine (5 equiv.) were dissolved in methanol-*d*_4_ (0.6 mL) to form [Ir(H)_2_(IMes)(pyridine)_3_]Cl. *T*_2_ and *D* were recorded using standard 1D CPMG and DOSY measurements respectively. These revealed *T*_2_ times for the *ortho*, *meta* and *para* pyridine sites of 701, 724 and 868 ms respectively, which are compressed relative to the 1123, 1324 and 931 ms for these sites recorded without the metal catalyst present. In contrast, there was no effect on the self-diffusion coefficient of pyridine which remained as *D =* 1.6 × 10^−9^ m^2^ s^−1^. This shortening of pyridine *T*_2_ can also be detected from UF LNMR. For example, when *D*–*T*_2_ sequences (containing a CPMG loop) are used to examine this solution, extracted *T*_2_ values shorten from 870 ± 330 ms (for 25 mM pyridine) to 546 ± 122 ms upon addition of the metal precatalyst.

This solution was then degassed and activated with 3 bar pH_2_ overnight to form the SABRE-active [Ir(H)_2_(IMes)(pyridine)_3_]Cl. During this process, *T*_2_ and *D* values were recorded using both 1D CPMG and DOSY sequences and UF LNMR (*D*–*T*_2_ containing a CPMG loop). Interestingly, both traditional Fourier-transform NMR and ultrafast Laplace NMR reveal a further drop in *T*_2_ as [Ir(COD)(IMes)(pyridine)]Cl is converted into [Ir(H)_2_(IMes)(pyridine)_3_]Cl, and is expected due to the binding of two more pyridine ligands per metal centre. However, a slight increase in *T*_2_ at longer reaction times (Fig. 5b and c) is observed by both traditional NMR and ultrafast Laplace NMR. It is well-known that iridium complexes of this type can catalyse hydrogen isotope exchange reactions in which deuterium atoms in the methanol solvent are exchanged with proton sites in H_2_ and pyridine.^40,45,49,52^ This results in the formation of HD and deuterated-pyridine. We confirm from ^1^H NMR measurements that such effects are occurring in this reaction. For example, prior to hydrogen addition pyridine *ortho*, *meta* and *para* sites were observed with a signal integral intensity of 1 : 1 : 0.6 which decreased to 0.3 : 1 : 0.6 after being left under 3 bar H_2_ overnight (Fig. 5d). This loss of pyridine *ortho* signal reflects the deuteration of *ca.* 60% pyridine *ortho* sites. The appearance of a 1 : 1 : 1 triplet at *δ* 4.54 ppm is characteristic of HD,^47^ which further supports that such a hydrogen isotope exchange reaction has occurred here. We expect that these effects account for the increase in pyridine *T*_2_ observed at longer reaction times and note that similar effects have been reported for *T*_1_ behaviour with similar substrates in related systems.^49^ It is clear that changes in *T*_2_, determined from both 1D CPMG and 2D UF LNMR sequences, can also be used to gain information on the chemical reactions occurring in this activation process.

It is also worth noting that a slight (*ca.* 8%) drop in *D* for free pyridine is observed using DOSY experiments which is associated with pyridine binding to the metal centre during the mixing time of these experiments (200 ms). The rate of pyridine dissociation from [Ir(H)_2_(IMes)(pyridine)_3_]Cl has been reported to be 23 s^−1^ at 300 K,^53^ therefore we expect around 4–5 exchange events to occur within the 200 ms diffusion delay used in these DOSY experiments, reflecting an intermediate exchange regime. We can also detect this decrease in *D* using UF LNMR experiments (Fig. 4c). Here, we observe a lower average decrease in pyridine diffusion coefficient (*ca.* 3%) which is likely related to fewer exchange events (*ca.* 1) within the shorter 50 ms diffusion delay of Laplace NMR experiments.

Laplace NMR has been used to monitor chemical processes that result in changes in *T*_1_, *T*_2_ or *D*,^54,55^ although the technique is most typically applied to detection of bulk water at M concentrations.^36,56,57^ Here, monitoring of chemical reactions involving species at tens of mM concentrations using ultrafast LNMR has been demonstrated. The advantage of using Laplace NMR to monitor these reactions is that the experiments used here took just 11 minutes and could provide information on both *D* and *T*_2_ in the same measurement, whereas 1D CPMG and DOSY experiments took 36 min each, providing a *ca.* 6.5-fold time saving.

### Improving sensitivity of UF LNMR by using SABRE hyperpolarisation

**Fig. 6:**
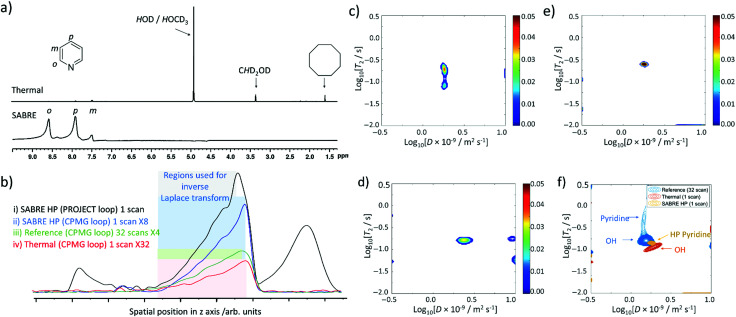
SABRE hyperpolarised ultrafast Laplace NMR. Single scan (a) ^1^H NMR spectra (b) excitation detection profiles and (c–e) *D*–*T*_2_ correlation spectra recorded after a solution containing [IrCl(COD)(IMes)] (5 mM) and pyridine (5 equiv.) in methanol-*d*_4_ (0.6 mL) are shaken with 3 bar pH_2_ for 10 seconds at 65 G. In (a) the thermally polarised single scan ^1^H NMR spectra is also shown above for comparison. (c) and (d) were recorded using a CPMG loop with a 0 and 3 second delay between sample insertion and spectral acquisition respectively. (e) was recorded using a PROJECT loop with a 0 second delay. (f) shows the spectra from (e) (yellow) overlaid with single scan (orange) and 32 scan (blue) thermally polarised reference spectra. (c–e) were recorded in 3 seconds which provides an overall 1440-fold time saving compared to measurement of *D* and *T*_2_ from conventional NMR (36 min each).

Even though ultrafast Laplace NMR experiments can measure relaxation and diffusion parameters with experiment times reduced by 1 to 3 orders of magnitude compared to Fourier transform NMR, or standard Laplace NMR, they still suffer from low thermal polarisation.^2^ Therefore, hyperpolarised Laplace NMR can be highly beneficial to boost the signal intensity of single scan measurements.^2,20,21,36^ For the first time we report the combination of Laplace NMR with the SABRE hyperpolarisation technique. Samples containing the active [Ir(H)_2_(IMes)(pyridine)_3_]Cl SABRE catalyst were prepared as previously described and shaken manually with fresh 3 bar pH_2_ for 10 seconds in a 65 G magnetic field before insertion into the 9.4 T spectrometer. This 65 G field is essential to match the resonance conditions required to transfer magnetisation from the pH_2_-derived hydride ligands to pyridine ^1^H sites within [Ir(H)_2_(IMes)(pyridine)_3_]Cl.^24–27,29,30^ In our studies these fields are generated by an electromagnetic coil which is described in the ESI† (Fig. S11). Upon pH_2_ shaking and insertion into the magnet, single scan ^1^H NMR spectra were collected to confirm that pyridine ^1^H NMR signals were enhanced by SABRE (Fig. 6a). These spectra revealed pyridine ^1^H NMR signals enhanced by a factor of 307-, 13-, and 145-fold for the *ortho*, *meta* and *para* sites respectively. Under these hyperpolarised conditions pyridine signals are now 62 times larger than those of *H*OD/*H*OCD_3_ at *δ* 5.25 ppm whereas in single scan thermally polarised spectra they are 9 times weaker (Fig. 6a).

At this point, fresh pH_2_ was added, the shaking process repeated, and single scan ultrafast *D*–*T*_2_ Laplace NMR sequences were collected. The intensity of the spectral profile is much larger for SABRE HP measurements compared to analogous single scan *D*–*T*_2_ sequences recorded using thermal polarisation (Fig. 6b). *D*–*T*_2_ correlation spectra (containing either CPMG or PROJECT loops) reveal the presence of a dominant peak (Fig. 6c–e) which is expected to correspond to pyridine, whose ^1^H NMR signals dominate the single scan ^1^H NMR spectra (Fig. 6a). This signal contains *D* and *T*_2_ values of 1.7 ± 0.3 × 10^−9^ m^2^ s^−1^ and 236 ± 44 ms respectively which reflects a shortening of *T*_2_ relative to those of pyridine determined from multi-scan thermally polarised Laplace NMR measurements (Fig. 6f). The effect of factors such as diffusion upon *T*_2_ encoding, which can shorten apparent *T*_2_ values determined from LNMR compared to those determined using traditional methods, have already been highlighted. A further apparent *T*_2_ shortening in single scan SABRE HP UF LNMR measurements compared to many-scan thermally polarised UF LNMR can be related to temperature effects caused by preparation of the hyperpolarised state at *ca.* 291 K outside the magnet before rapid transfer to the spectrometer at 298 K.

The *D*–*T*_2_ correlation spectra shown in Fig. 6c–e are reproducible with similar spectra obtained when a variable time delay (1 or 2 s) is left between insertion of the HP sample into the magnet and spectral acquisition (see ESI,† Section S5.1). This delay was introduced to examine the effect of potential movement of the solution on the appearance of these spectra if recorded immediately upon sample insertion. *D*–*T*_2_ correlation spectra appear clearer with less background noise/artefacts when sequences using PROJECT loops are used (Fig. 6e). Similar improvements in spectral clarity are observed when a 3 second time delay is left between sample insertion and spectral acquisition (Fig. 6d).

When analogous single scan thermally polarised *D*–*T*_2_ correlation spectra are collected, only a signal assigned as O*H* from *H*OD/*H*OCD_3_ can be observed. Therefore, there is a clear benefit to the use of SABRE hyperpolarisation in ultrafast Laplace NMR experiments as it can make minor components (in this case pyridine) visible that would otherwise require greater signal averaging (and an associated time investment) to be observed. They can also provide a route to remove signals for non-hyperpolarised molecules, in this case background solvent, which may be visible in single scan thermally polarised measurements. This may prove valuable in using SABRE HP Laplace NMR for the detection of low concentration molecules in the presence of a background matrix.

## Conclusions

In conclusion, we have demonstrated the use of ultrafast Laplace NMR *D*–*T*_2_ correlation sequences to measure molecular self-diffusion coefficients (*D*) and transverse relaxation times (*T*_2_) for pyridine (5 M and 25 mM) in methanol-*d*_4_. *D*–*T*_2_ correlation spectra often contain additional signals arising from background noise and *J*-coupled artefacts, although these can be removed by replacing CPMG loops with PROJECT loops in ultrafast Laplace NMR *D*–*T*_2_ pulse sequences. For the first time SABRE hyperpolarisation has been used in combination with ultrafast Laplace NMR detection and it can measure *D* coefficients of pyridine at a 25 mM concentration in just 3 seconds. These values are consistent with those recorded using DOSY which take significantly longer to acquire (36 minutes). This reflects a 720-fold improvement in time saving and has implications for rapid determination of *D* for a range of other SABRE hyperpolarised molecules. *T*_2_ can also be determined using ultrafast Laplace NMR (providing an overall time saving of 1440-fold compared to conventional NMR when both *D* and *T*_2_ are measured), although extracted *T*_2_ values are often shorter than real *T*_2_ determined from 1D CPMG experiments. Nevertheless, relative changes in *T*_2_ can be discerned and we provide an example of using ultrafast Laplace NMR for reaction monitoring by tracking pyridine *T*_2_ changes linked to iridium ligation and deuteration.

It is more common for *T*_1_ relaxation times in hyperpolarised systems to be studied as this describes the time taken for enhanced magnetisation to relax back to its thermally polarised state. This value therefore gives a useful indication of the timescale over which hyperpolarised states may last. In contrast, studies involving *T*_2_ of SABRE HP-systems are rarer and are expected to become more common as progress towards biomedical imaging applications are made. This work presents valuable information on substrate *T*_2_ and how this can be influenced by metal binding and deuteration in a manner mirroring *T*_1_. In the future, ultrafast Laplace NMR sequences could be developed that allow the determination of *T*_1_. Sequences that measure *T*_1_–*T*_2_ correlation have already been reported,^3,58^ but these must be modified for use in a hyperpolarised single scan approach.

SABRE hyperpolarised NMR has already been used to monitor reactions based upon changes in substrate chemical shift.^59–61^ Laplace NMR can be useful for monitoring processes in which a substrate undergoes a change in *T*_2_ or *D*, particularly in cases where there may be no discernible change in chemical shift. This approach may be particularly useful to monitor rapid catalyst activations (for example, those of sulfoxide-based SABRE catalysts^33,34,47,62^) or even catalyst dimerisation that may be accompanied by a large change in *D*.^34,61,63^ This approach may be of particular relevance for reactions studied at low, ultra-low, or even zero field.^64,65^ It is also possible to use sequences with selective chirp pulses to excite only the pyridine resonance(s). This may limit the appearance of additional background solvent signals in *D*–*T*_2_ correlation spectra. We have chosen not to employ these sequences currently as we envision the use of SABRE HP Laplace NMR for the study and identification of molecules in complex mixtures for which there may be many different analytes with similar chemical shift, but different *T*_1_, *T*_2_ or *D*.

## Author contributions

BJT: conceptualisation, investigation, validation, visualisation, writing – original draft; VVZ: conceptualisation, funding acquisition, investigation, resources, pulse sequences, processing software, creating setups, synthesis of [IrCl(COD)(IMes)] precatalyst, supervision, validation, writing – review and editing; VVT: conceptualisation, funding acquisition, resources, supervision, validation, writing – review and editing.

## Conflicts of interest

There are no conflicts to declare.

## Supplementary Material

CP-023-D1CP02383G-s001
