# Supplementary material for: Ultrafast Laplace NMR to study metal–ligand interactions in reversible polarisation transfer from parahydrogen
Source: Phys Chem Chem Phys. 2021 Jul 22;23(31):16542–50. doi: 10.1039/d1cp02383g (PMC8359933; doi:10.1039/d1cp02383g)
Supplement: CP-023-D1CP02383G-s001 [file CP-023-D1CP02383G-s001.pdf]

Electronic Supplementary Material (ESI) for Physical Chemistry Chemical Physics.
This journal is © the Owner Societies 2021

Supporting Information

Ultrafast Laplace NMR to study metal-ligand interactions in reversible polarisation transfer from parahydrogen

Ben. J. Tickner,[+a] Vladimir V. Zhivonitko[+*a] and Ville-Veikko Telkki*[a]

[a] NMR Research Unit, Faculty of Science, University of Oulu, P.O. Box 3000, 90014 Oulu, Finland.

[+] These authors contributed equally

* Corresponding authors: vladimir.zhivonitko@oulu.fi; ville-veikko.telkki@oulu.fi

Table of Contents

S1. Using ultrafast Laplace NMR to measure *D* and T_2 of concentrated thermally polarised pyridine

S2. Using ultrafast Laplace NMR to measure *D* and T_2 of dilute solutions of thermally polarised pyridine

S2.1 Effect of number of scans on ultrafast Laplace NMR *D*-T_2

S2.2 Effect of excitation frequency on ultrafast Laplace NMR *D*-T_2

S3. Effect of *J*-modulation on 1D CPMG data

S4. Formation of SABRE catalysts examined using thermally polarised ultrafast Laplace NMR

S5. Improving MR sensitivity of UF LNMR by using SABRE hyperpolarisation

S5.1 Effect of delayed acquisition on SABRE HP ultrafast Laplace NMR *D*-T_2

S5.2 Reproducibility of SABRE HP ultrafast Laplace NMR *D*-T_2

S6. References

S1. Using ultrafast Laplace NMR to measure D and T_2 of concentrated thermally polarised pyridine

A solution of pyridine (5 M) in methanol-d_4 (0.6 mL) was used for determination of D and T_2 using ultrafast LNMR D-T_2 pulse sequences. D-T_2 correlation spectra are shown in Figure S1a. Analogous measurements on a reference sample of methanol-d_4 (0.6 mL) are shown in Figure S1b. A D-T_2 correlation spectrum for a sample containing pyridine (5 M) in methanol-d_4 (0.6 mL) recorded using D-T_2 UF LNMR sequences with a PROJECT loop is shown in Figure S1c. Overlaid D-T_2 spectra are shown in Figure S1d.

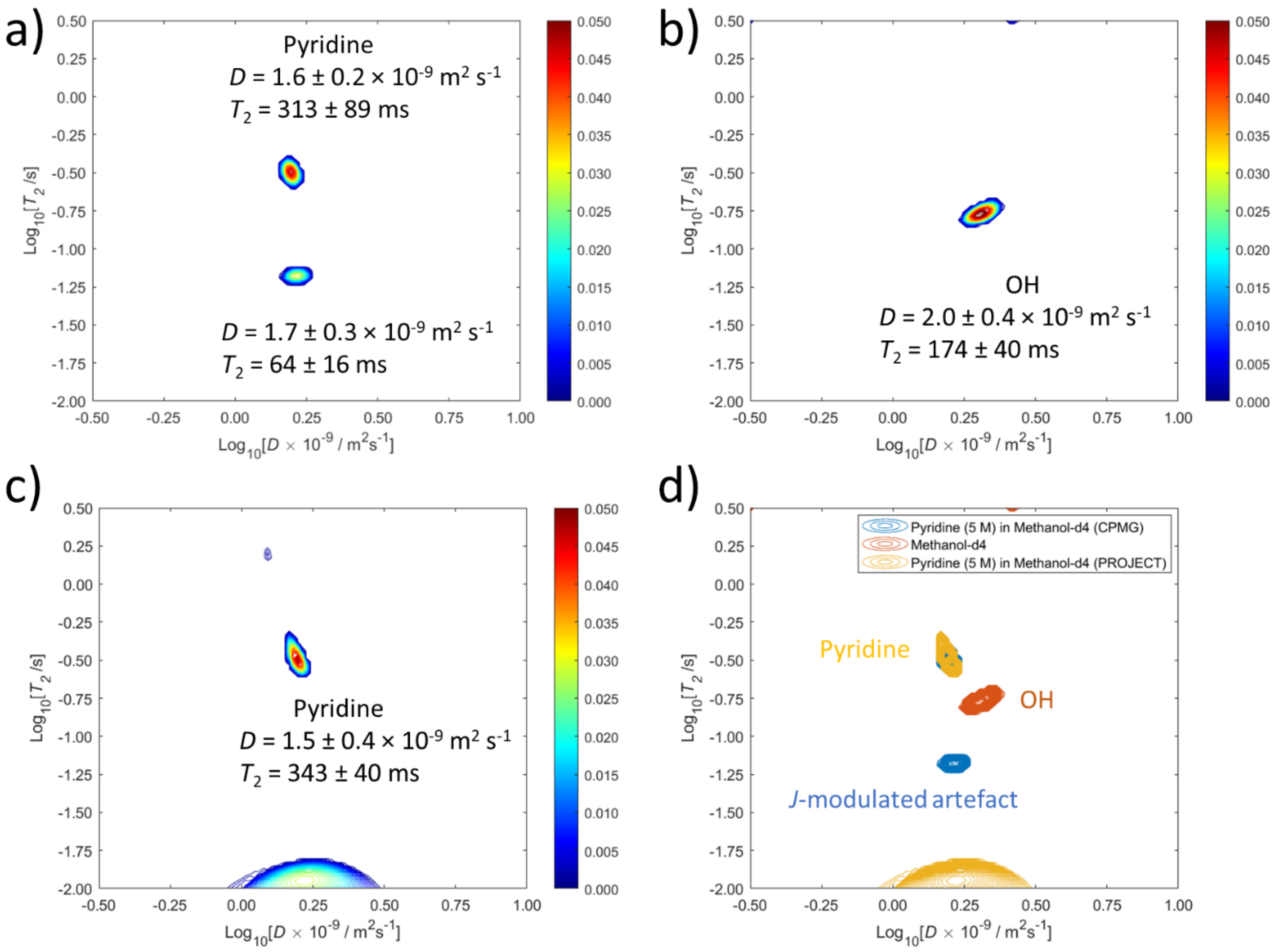

Figure S1: Thermally polarised a) D-T_2 correlation (with CPMG loop) of pyridine (5 M) in methanol-d_4 b) D-T_2 correlation (with CPMG loop) of methanol-d_4 (0.6 mL) c) D-T_2 correlation (with PROJECT loop) for pyridine (5 M) in methanol-d_4 d) overlaid D-T_2 UF LNMR spectra. Note that Figure S1a and d are shown in the main paper.

S2. Using ultrafast Laplace NMR to measure *D* and T_2 of dilute solutions of thermally polarised pyridine

A solution of pyridine (25 mM) in methanol-d_4 (0.6 mL) was used for determination of *D* and T_2 using ultrafast LNMR *D*-T_2 pulse sequences. *D*-T_2 correlation spectra are shown in Figure S2a. A *D*-T_2 correlation spectrum recorded using *D*-T_2 UF LNMR sequences with a PROJECT loop is shown in Figure S2b. Overlaid *D*-T_2 spectra are shown in Figure S2c. This figure also contains an overlap with analogous measurements on a reference sample of methanol-d_4 (0.6 mL), the which are shown in Figure S1b (with a CPMG loop).

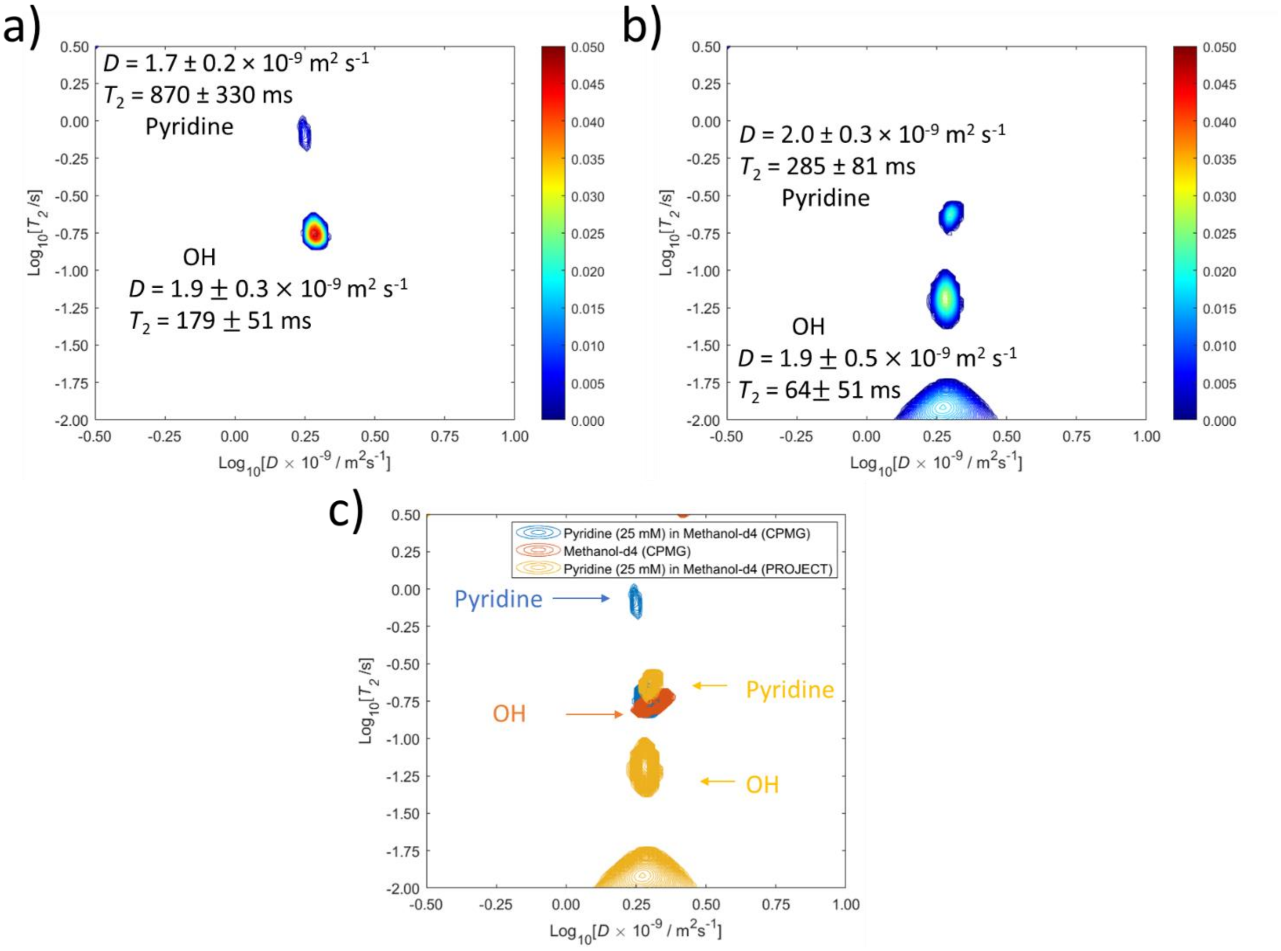

Figure S2: Thermally polarised a) *D*-T_2 correlation (with CPMG loop) and b) *D*-T_2 correlation (with PROJECT loop) of pyridine (25 mM) in methanol-d_4. c) overlaid *D*-T_2 spectra. Note that Figure S2a and c are shown in the main paper.

S2.1 Effect of number of scans on ultrafast Laplace NMR *D*-T_2

The effect of the number of scans on *D*-T_2 correlation spectra for a solution of pyridine (25 mM) in methanol-d_4 (0.6 mL) was determined by using ultrafast LNMR *D*-T_2 pulse sequences with 1, 4, 32 and 256 scans. In *D*-T_2 correlation spectra only the *OH* signal from *HOD* / $HOCD_3$ can be resolved using 1 scan (Figure S3a). When 4 scans are used the minor pyridine component can now be resolved, but its *D* and T_2 values are unreliable (Figure S3b). 32 scans are sufficient to generate reliable data (Figure S3c) which is improved when greater number of scans are used (Figure S3d).

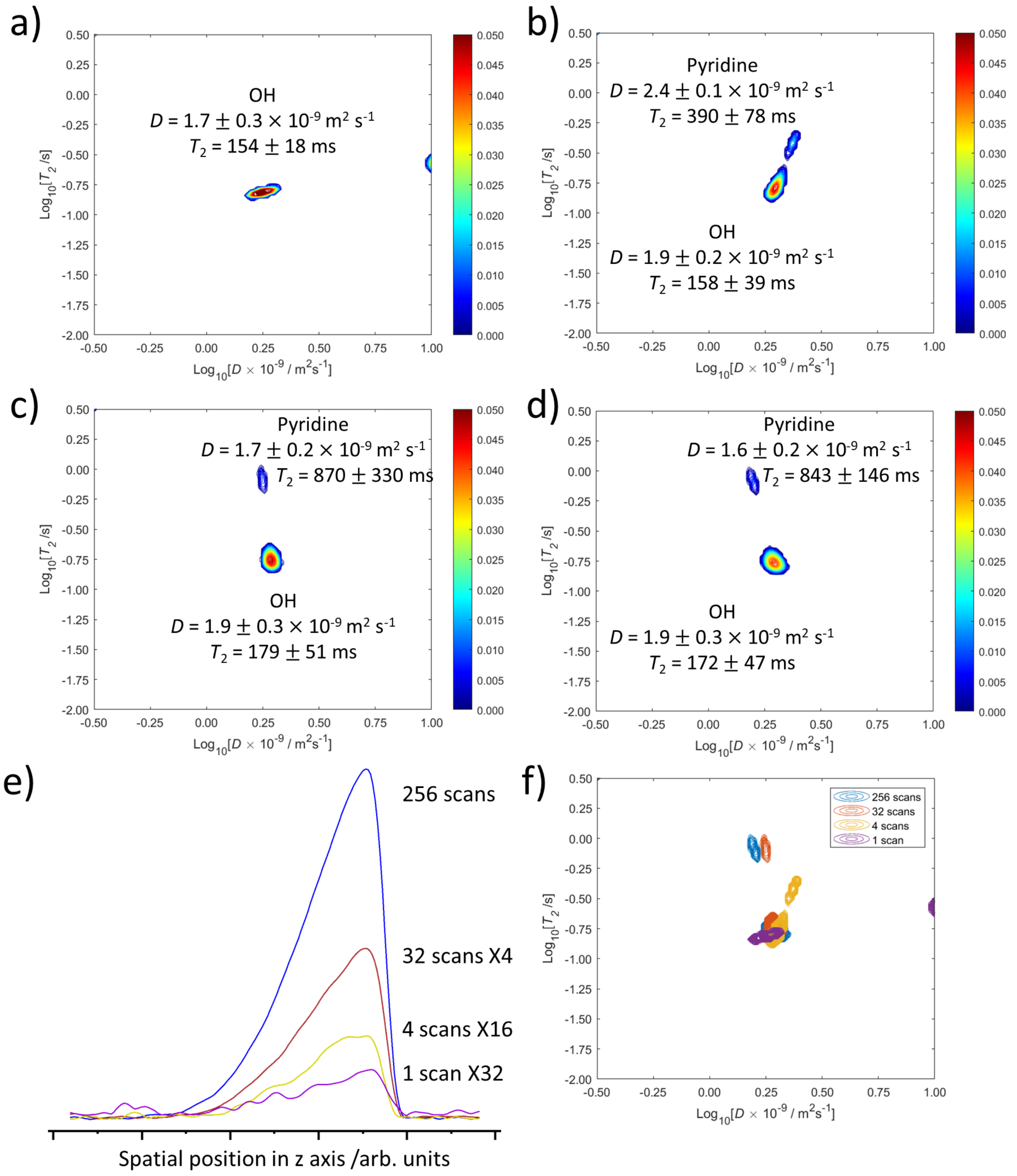

Figure S3: Thermally polarised *D*-T_2 correlation (with CPMG loop) for a sample of pyridine (25 mM) in methanol-d_4 recorded using a) 1 scan (22 seconds) b) 4 scans (1 minutes 25 seconds) c) 32 scans (11 minutes, 13 seconds) and d) 256 scans (1 hour, 29 minutes and 42 seconds). e) Overlaid spectral profiles used to generate the *D*-T_2 correlation plots shown in a)-d). Note that they are not all shown to the same vertical scale, the magnification is given in the legend. f) Overlaid *D*-T_2 UF LNMR spectra.

S2.2 Effect of excitation frequency on ultrafast Laplace NMR D-T_2

The effect of the central excitation frequency (o1p) on D-T_2 correlation spectra for a solution of pyridine (25 mM) in methanol-d_4 (0.6 mL) was determined by using ultrafast LNMR D-T_2 pulse sequences with a varying o1p parameter. In all experiments presented in this work the o1p was set to 6.378 ppm. We expect the excitation pulses to excite all protons within the δ = 0 to 10 ppm range. Nevertheless, we investigated the effect of changing o1p on the experimental results. D-T_2 correlation spectra recorded with an o1p set at 1.000 ppm (noise) and 8.570 ppm (pyridine *ortho* resonance) are shown in Figure S4a and S4b respectively. Figure S4c shows that when overlapped, spectra appear similar, but D and T_2 values that are extracted can change depending on the o1p value used.

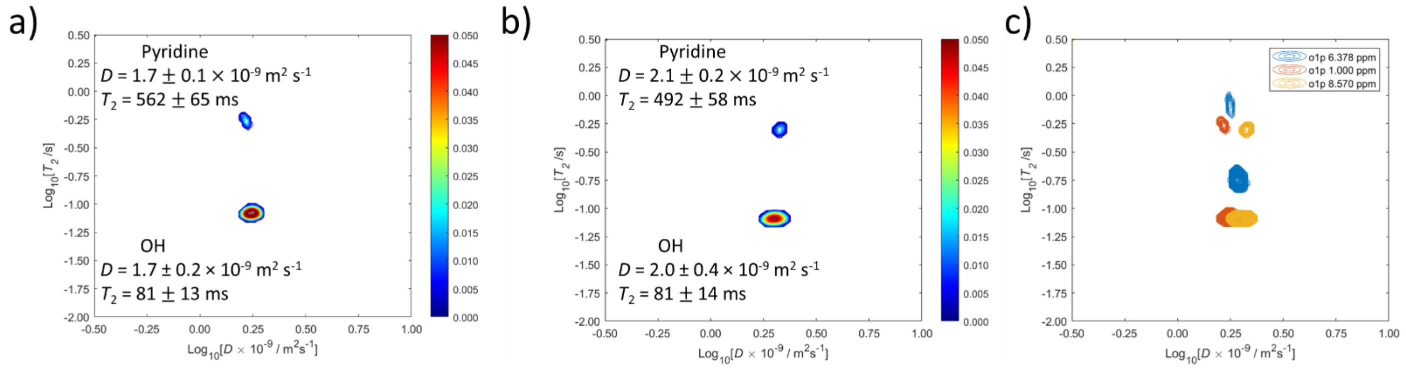

Figure S4: Thermally polarised D-T_2 correlation (with CPMG loop) for a sample of pyridine (25 mM) in methanol-d_4 recorded using an o1p set to a) 1.000 ppm and b) 8.570 ppm. Spectra overlaid with those collected using an o1p of 6.378 ppm is shown in c).

The effect of changing o1p on D-T_2 correlation spectra collected using a PROJECT loop in the pulse sequence (instead of a CPMG loop) is shown in Figure S5 and appears to be less influenced by the o1p value.

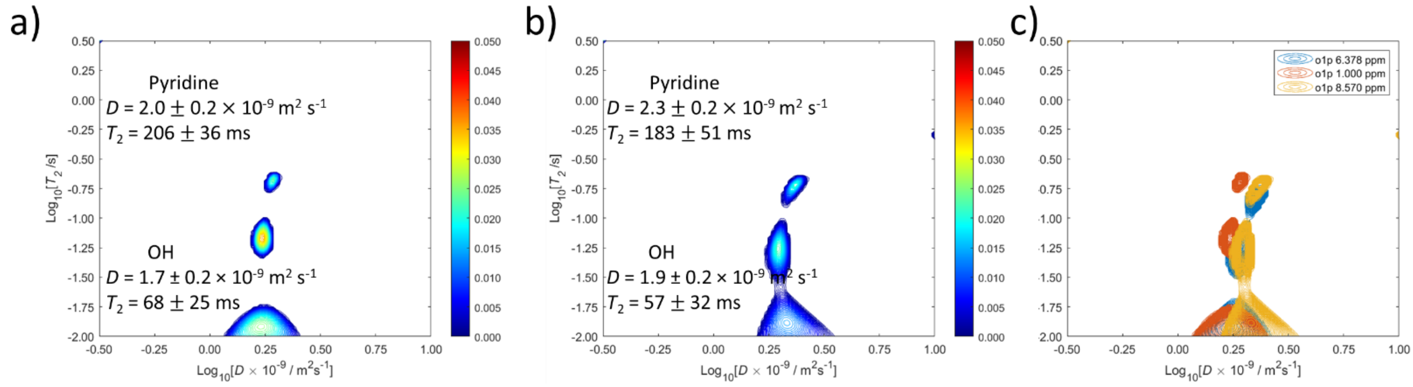

Figure S5: Thermally polarised D-T_2 correlation (with PROJECT loop) for a sample of pyridine (25 mM) in methanol-d_4 recorded using an o1p set to a) 1.000 ppm and b) 8.570 ppm. Spectra overlaid with those collected using an o1p of 6.378 ppm is shown in c).

S3. Effect of *J*-modulation on 1D CPMG data

Ultrafast *D*-T_2 sequences recorded using a CPMG loop can give rise to *J*-modulated artefacts (*e.g.* Figure S1a). Related effects can also be observed in 1D CPMG data recorded using a short echo time (4 ms) (Figure S6).

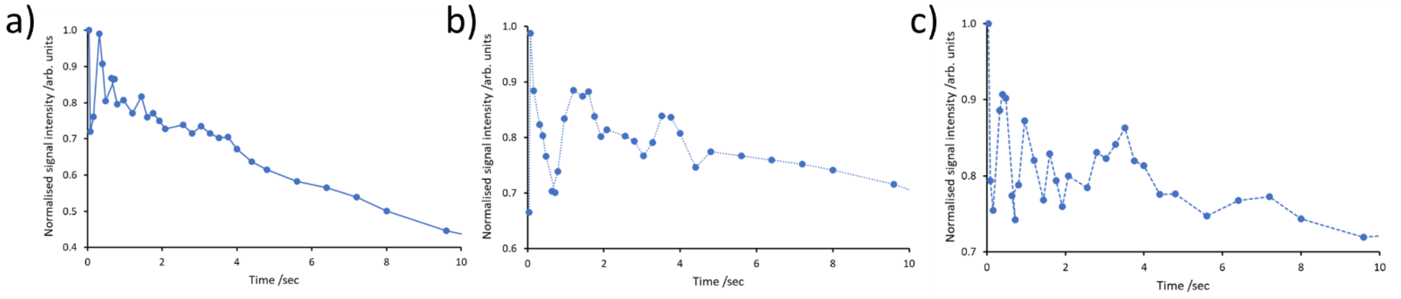

Figure S6: Example ^{1}H NMR signal intensity for the pyridine a) *ortho* b) *meta* and c) *para* signals as a function of evolution time measured from 1D CPMG spectra for a sample of pyridine (25 mM) in methanol-*d*$_4$. The dashed line is to aid visual interpretation and does not reflect a T_2 fitting curve. The echo time used was 4 ms.

These effects can be reduced by using longer echo times (15 ms) (Figure S7). We confirm that both 1D T_2 data recorded using a CPMG or a PROJECT sequence with a 15 ms echo time (30 ms double spin echo time in the case of the PROJECT sequence) are consistent (Figure S7). We note that for this T_2 data recorded using a 15 ms echo time the pyridine *meta* site still appears to show the effects of *J*-modulation and this is likely responsible for the larger differences observed between PROJECT and CPMG sequences for this resonance (Figure S7c).

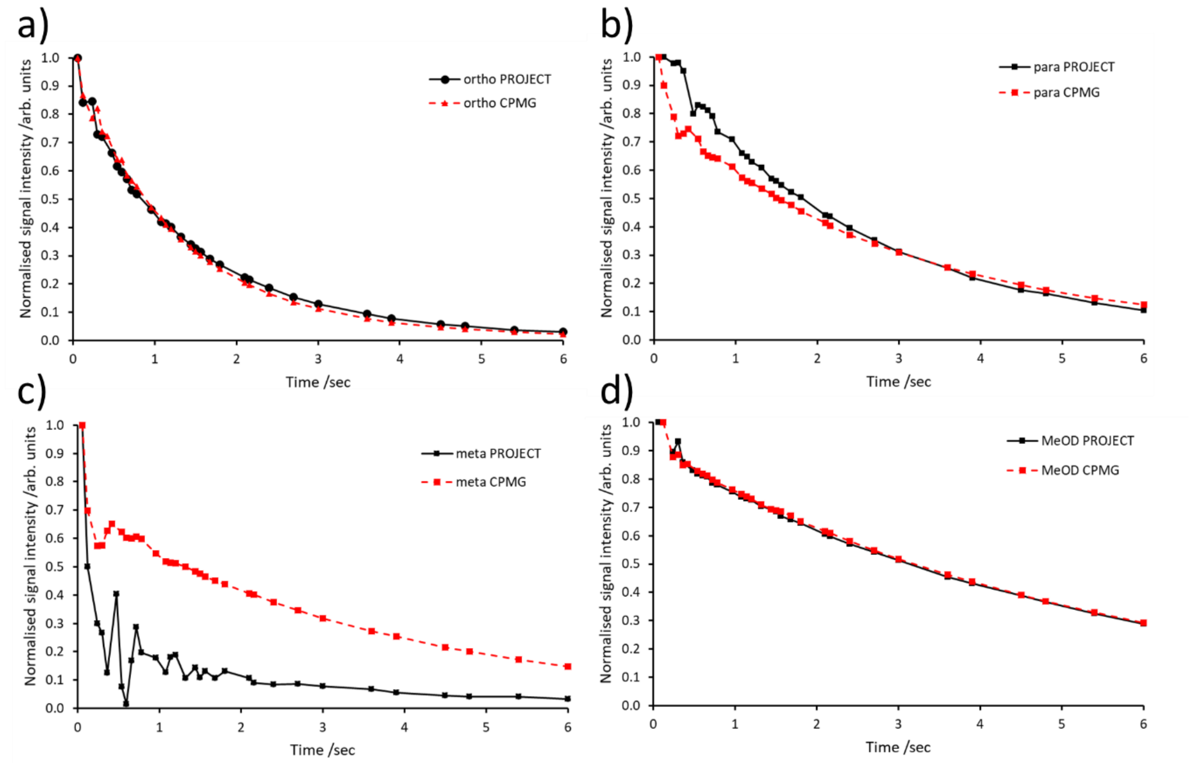

Figure S7: Example ^{1}H NMR signal intensity for the a) pyridine *ortho* b) pyridine *para* c) pyridine *meta* and d) C*H*CD_2OD signal as a function of evolution time measured from 1D CPMG (red dashed) and PROJECT (black solid) spectra for a sample of pyridine (25 mM) in methanol-*d*$_4$. The lines are to aid visual interpretation and do not reflect T_2 fitting curves. The echo time used was 15 ms (30 ms double spin echo in the case of the PROJECT sequences).

While it has been reported that the effect of *J*-modulation is generally more pronounced when longer echo times are used,[1] this was not visible when 1D CPMG data was collected using a 40 ms echo time (Figure S8). Here, experiments with longer echo times record data points at sufficient time spacings that the majority of 1D CPMG data recorded with 40 ms echo times produce signal decay plots that do not exhibit *J*-modulated oscillations. They can therefore be fit to a mono-exponential decay function to yield a reliable T_2. Any data containing oscillations were discarded and a T_2 value was not extracted.

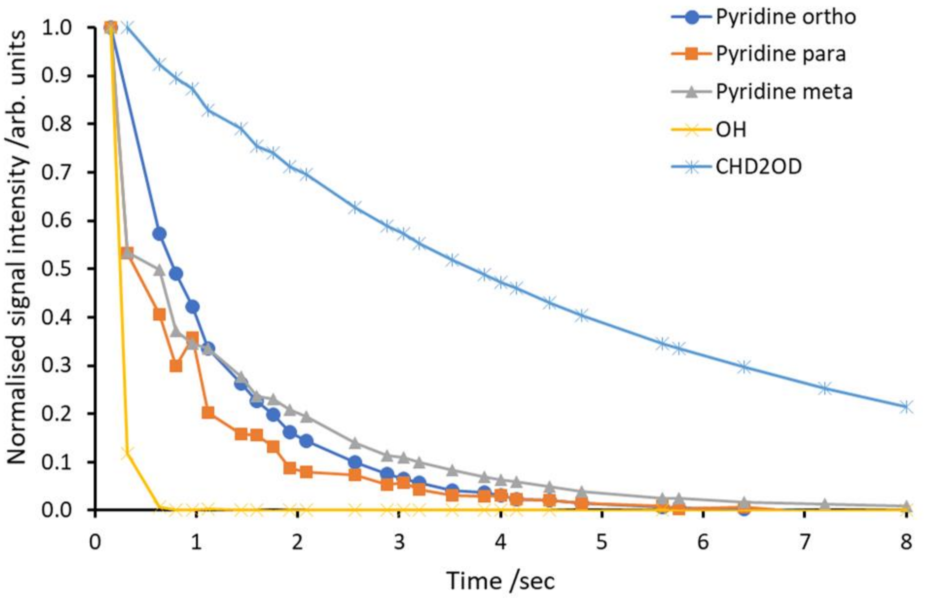

Figure S8: Example ^{1}H NMR signal intensities for the pyridine, O*H* and C*H*D$_2$OD resonances as a function of evolution time measured from 1D CPMG spectra for a sample of pyridine (25 mM) in methanol-*d*$_4$. The lines are to aid visual interpretation and do not reflect a T_2 fitting curve. The echo time used was 40 ms.

S4. Formation of SABRE catalysts examined using thermally polarised ultrafast Laplace NMR

Hydrogen gas was produced from the electrolysis of water using a desktop hydrogen generator (F-DGSi, Evry, France). This was used directly to make parahydrogen (pH_2) using a BPHG 90 parahydrogen generator (Bruker) which passes the hydrogen gas over a spin-exchange catalyst at low temperature. The generator operates at *ca* 38 K and produces a constant flow of pH_2 with *ca* 92% purity. Parahydrogen (3 bar) was added to NMR tubes containing a quick pressure valve using a home-built system shown in Figure S9. This set-up contains 3 valves that allow the system to be opened to A) the NMR tube B) parahydrogen and C) a vacuum pump. This system was used to degas the sample. Samples were left to react with H_2 (3 bar) for a period of several hours (usually around 16 hours overnight). Activation is usually indicated by a change in colour from orange to pale orange. Catalyst activation is indicated in ^{1}H NMR spectra by the formation of a peak corresponding to $[Ir(H)_2(IMes)(pyridine)_3]Cl$ at $\delta = -22.74$ ppm.[2] As catalyst activation proceeds, ^{1}H NMR signals for the COD ligand at $\delta = 3$ and 4 ppm eventually disappear and a signal for cyclooctane at *ca* $\delta = 1.6$ ppm becomes visible.[2–4]

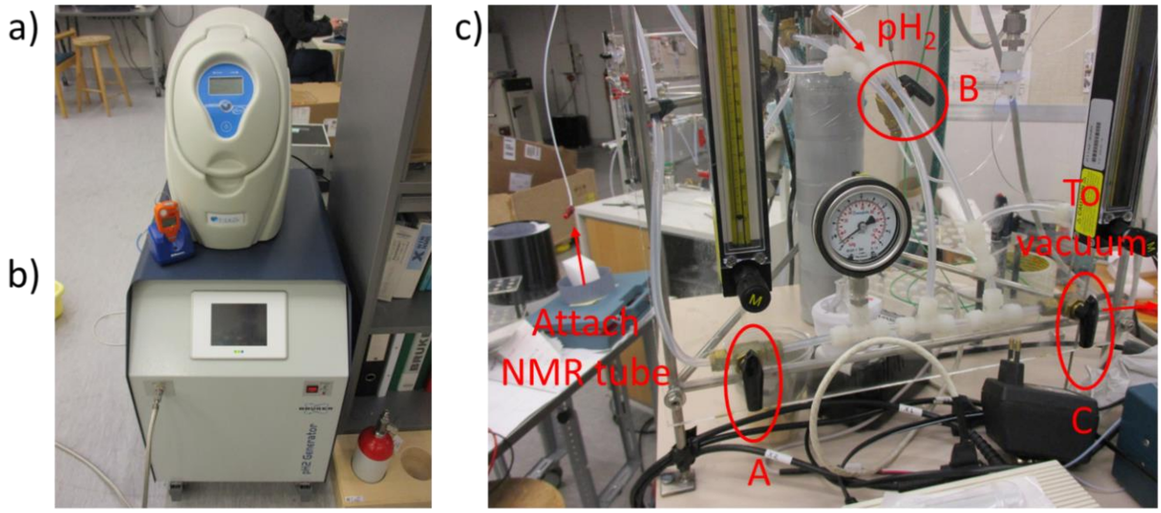

Figure S9: Picture of a) hydrogen generator b) parahydrogen generator and c) home-built device for addition of parahydrogen to NMR tubes in which A, B and C refer to the valves that allow opening of the system to the NMR tube, parahydrogen line or vacuum pump respectively.

A sample of [IrCl(COD)(IMes)] (5 mM) and pyridine (5 equiv.) were dissolved in methanol-d_4 (0.6 mL). This solution was then degassed and activated with 3 bar pH_2 overnight to form the SABRE-active $[Ir(H)_2(IMes)(pyridine)_3]Cl$. During this process, T_2 and D values were recorded using both 1D CPMG and DOSY sequences and UF LNMR (D-T_2 containing a CPMG loop). In some of these D-T_2 correlation plots a third signal may be discernible, in addition to those assigned as O*H* and pyridine. A representative spectrum containing this artefact peak with the largest intensity is shown in Figure S10a. This additional signal does not appear in measurements performed without the metal catalyst. This peak is often poorly resolved by the Laplace transform and corresponds to a diffusion coefficient of *ca* 7×10^{-9} m^2 s^{-1} and T_2 of *ca* 150 ms which do not show a consistent change over the activation period (Figure S10b-c). This larger D relative to pyridine and solvent makes this signal unlikely to correspond to species such as $[Ir(H)_2(IMes)(pyridine)_3]Cl$, which has a diffusion constant of *ca* 6×10^{-10} m^2 s^{-1} according to 1D DOSY measurements. Assignment due to other molecules present in these mixtures, such as cyclooctane, which is formed during catalyst activation,[3,5] is also unlikely as 1D DOSY and CPMG measurements reveal D and T_2 values of 1.5×10^{-9} m^2 s^{-1} and *ca* 3 s respectively for this molecule in these mixtures. T_2 values for $[Ir(H)_2(IMes)(pyridine)_3]Cl$ can not be determined using 1D CPMG sequences due to low signal intensity and peak overlap. It is most likely that this additional signal is an artefact that results from spectral noise or homonuclear J-coupling.[6–8]

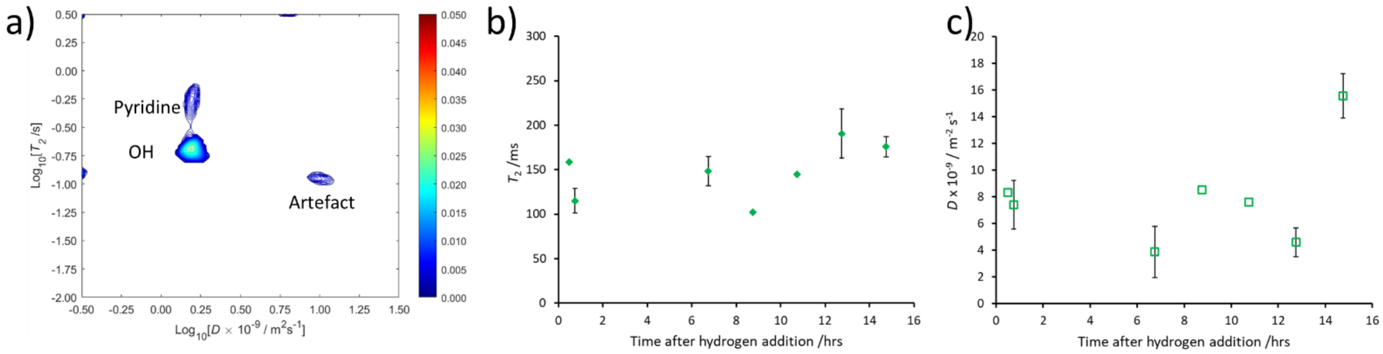

Figure S10: Thermally polarised a) D-T_2 correlation plot for a solution of [IrCl(COD)(IMes)] (5 mM) and pyridine (5 equiv.) in methanol-d_4 (0.6 mL) *ca* 12 hours after addition of H_2 (3 bar) at room temperature. Changes in b) T_2 and c) D of the artefact peak during catalyst activation measured using ultrafast Laplace NMR.

S5. Improving MR sensitivity of UF LNMR by using SABRE hyperpolarisation

Samples containing the active $[Ir(H)_2(IMes)(pyridine)_3]Cl$ SABRE catalyst were prepared by activating a solution containing [IrCl(COD)(IMes)] (5 mM) and pyridine (5 equiv.) in methanol-d_4 (0.6 mL) with H_2 (3 bar) overnight. This sample was then shaken manually with fresh 3 bar pH_2 for 10 seconds in a 65 G magnetic field before insertion into the 9.4 T spectrometer and collection of D-T_2 excitation-detection profiles. The shake and drop method was employed for recording hyperpolarised NMR spectra. This involves filling NMR tubes with fresh pH_2 (3 bar) as described in section S4 before shaking them vigorously for 10 seconds in a 6.5 mT (65 G) magnetic field.[2] We find that the stray field of our shielded 9.4 T magnet is no larger than 2 mT, therefore we use an electromagnetic coil powered by a Blanko PS-3005 0-30V 0-5A switching power supply to provide the necessary magnetic fields for SABRE polarisation transfer. The current and voltage of the power supply can be altered to achieve a magnetic field inside the coil of *ca* 6.5 mT. Magnetic fields were measured using a Hirst GM04 Gaussmeter and we estimate that the sample experiences a magnetic field of 6.5 ± 1.0 mT during the manual shaking process. This set up is shown in Figure S11 and is placed as close to the NMR spectrometer as possible to reduce transfer time after the 10 second shaking period. The sample is inserted into the spectrometer as rapidly as possible, this is facilitated by pre-emptively turning off the lift function on the spectrometer. ^1H NMR pulse sequences are modified to include an autosuspend function such that radiofrequency excitation occurs immediately upon sample insertion.

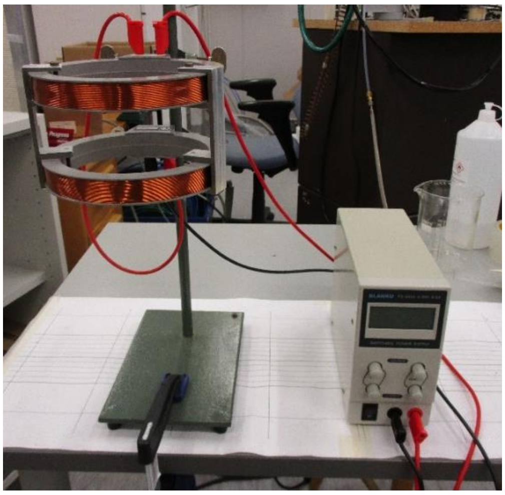

Figure S11: Picture of electromagnetic coil and power supply used to generate the 6.5 mT fields necessary for efficient SABRE polarisation transfer.

S5.1 Effect of delayed acquisition on SABRE HP ultrafast Laplace NMR *D*-T_2

D-T_2 Laplace NMR sequences containing a CPMG loop were used with a variable delay between insertion of the HP sample into the magnet and spectral acquisition. This delay was introduced to examine the effect of potential movement of the solution on the appearance of these spectra. (For example, at short delay times the solution may still be moving and this may influence spatial and/or diffusion encoding). The effect of this time delay is shown in Figure S12. There appears no significant difference between spectra collected with a 0, 1 or 2 second time delay, although spectral clarity does improve when a 3 second time interval is used. Generally, the intensity of the signal excitation detection profile appears to decrease as the delay time is increased which is consistent with a lower hyperpolarised ^1H NMR signal intensity due to greater relaxation as the delay is increased.

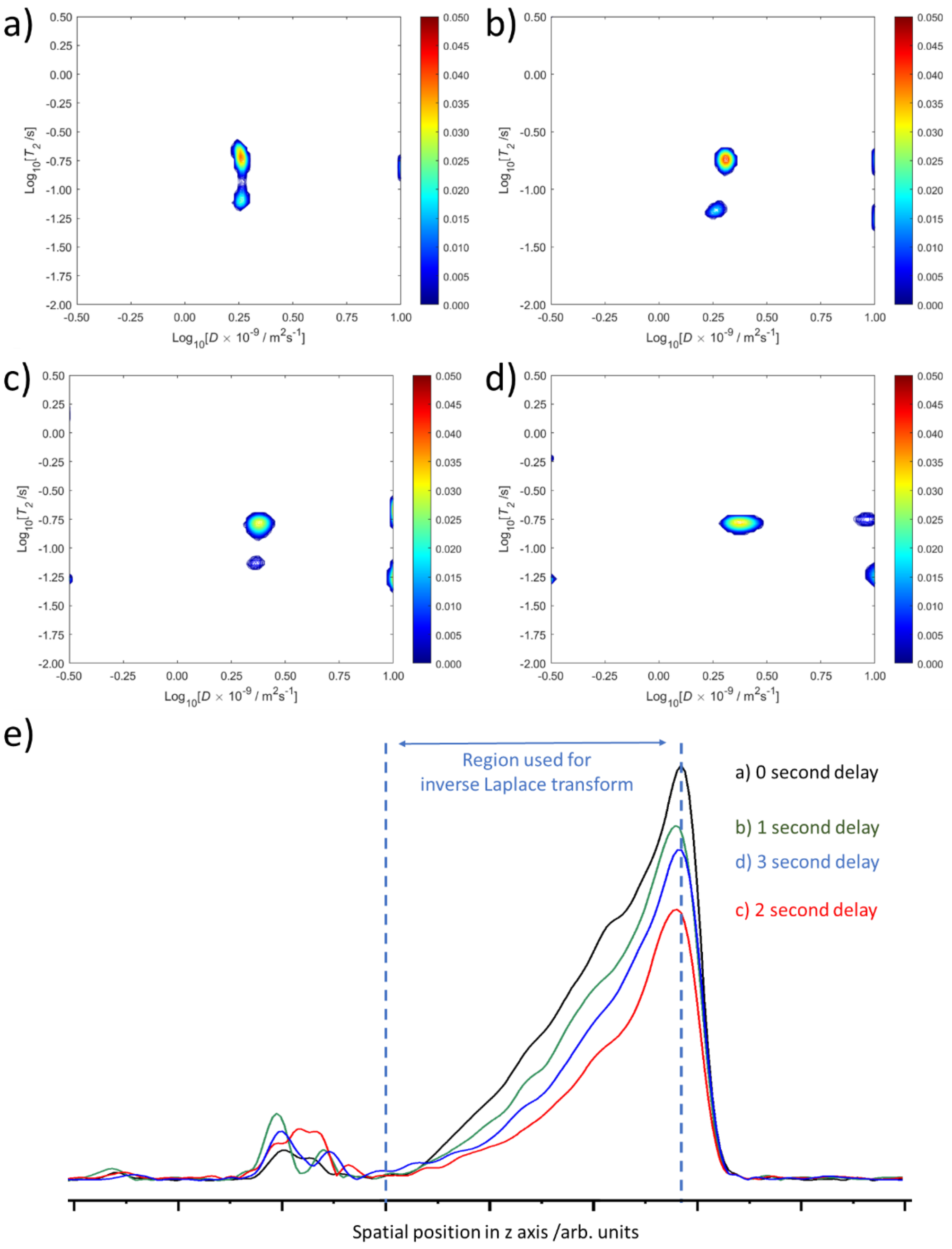

Figure S12: Example SABRE hyperpolarised *D*-T_2 correlation plots for a solution of [IrCl(COD)(IMes)] (5 mM) and pyridine (5 equiv.) in methanol-d_4 (0.6 mL) shaken with pH_2 (3 bar) for 10 seconds at 65 G with a a) 0 b) 1 c) 2 and d) 3 second time delay between sample insertion and spectral acquisition. Excitation detection profiles are shown in e).

S5.2 Reproducibility of SABRE HP ultrafast Laplace NMR *D*-T_2

The reproducibility of SABRE HP *D*-T_2 correlation spectra was investigated by repeating the shaking process with fresh pH_2 shaking on the same sample under the same conditions. These give similar *D*-T_2 correlation spectra (Figure S13) although the intensity of the excitation detection profile can change more significantly (*ca* 50 % difference) which is likely related to variation in ^{1}H NMR signal enhancements, although generally variation in the efficiency of SABRE polarisation transfer can be reduced by controlling more precisely the polarisation transfer field[9] or automated bubbling systems compared to manual shaking.[10–12]

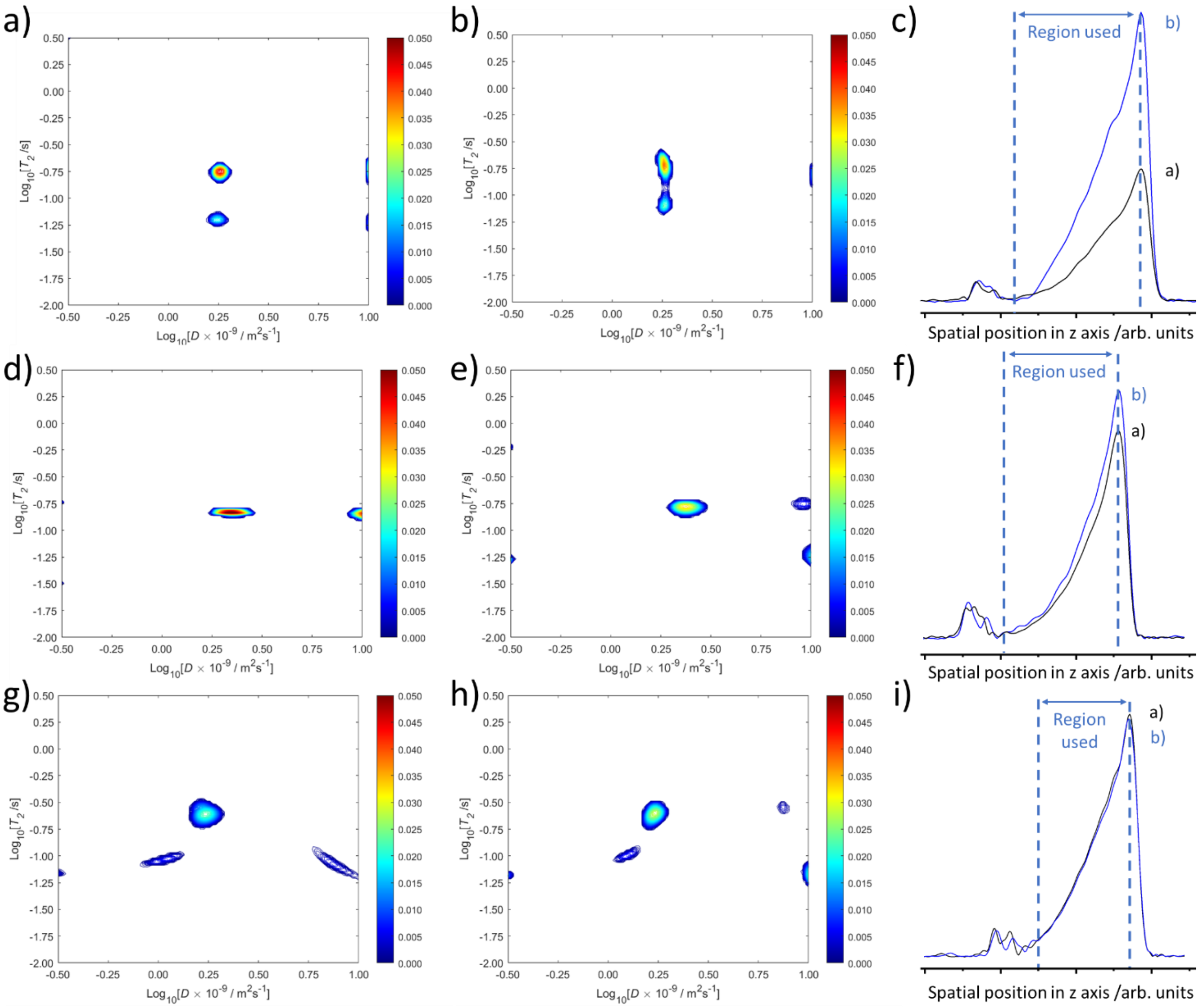

Figure S13: Comparison between repeated SABRE hyperpolarised *D*-T_2 correlation plots (CPMG loop) and spectral excitation detection profiles. a)-b) Example *D*-T_2 correlation plots for a solution of [IrCl(COD)(IMes)] (5 mM) and pyridine (5 equiv.) in methanol-d_4 (0.6 mL) shaken with pH_2 (3 bar) for 10 seconds at 65 G without a delay between sample insertion and spectral acquisition. c) overlaid spectral excitation detection profiles. d)-e) Example *D*-T_2 correlation plots when the solution from a)-c) is used and a 3 second time delay between sample insertion and spectral acquisition is used with f) analogous overlaid spectral excitation detection profiles. g)-h) Example *D*-T_2 correlation plots for a solution of [IrCl(COD)(IMes)] (5 mM) and pyridine (10 equiv.) in methanol-d_4 (0.6 mL) shaken with pH_2 (3 bar) for 10 seconds at 65 G without a delay between sample insertion and spectral acquisition. i) overlaid spectral excitation detection profiles.

The reproducibility of SABRE HP *D*-T_2 correlation spectra collected using a PROJECT loop was also investigated by repeating the shaking process with fresh *p*H_2 under the same conditions using the same sample. This yields consistent *D*-T_2 correlation spectra (Figure S14).

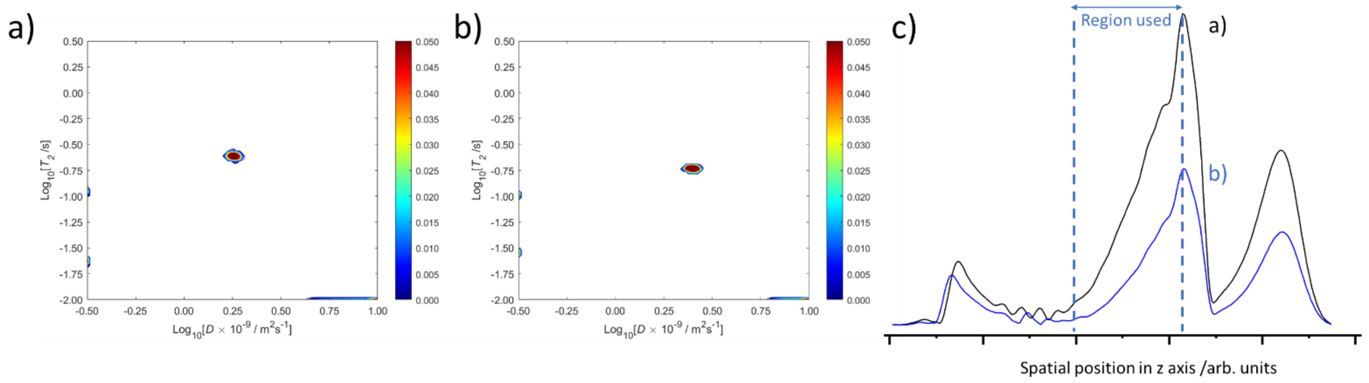

Figure S14: Comparison between repeated SABRE hyperpolarised *D*-T_2 correlation plots (PROJECT loop) and spectral excitation detection profiles. a)-b) Example *D*-T_2 correlation plots for a solution of [IrCl(COD)(IMes)] (5 mM) and pyridine (5 equiv.) in methanol-*d_4* (0.6 mL) shaken with pH_2 (3 bar) for 10 seconds at 65 G without a delay between sample insertion and spectral acquisition. c) overlaid spectral excitation detection profiles.

The reproducibility of SABRE HP *D*-T_2 (CPMG loop) correlation spectra collected using two separate samples both containing the same amount of SABRE precatalysts and substrate, hyperpolarised in the same manner and recorded using the same spectral acquisition parameters was also investigated and found to yield consistent results (Figure S15).

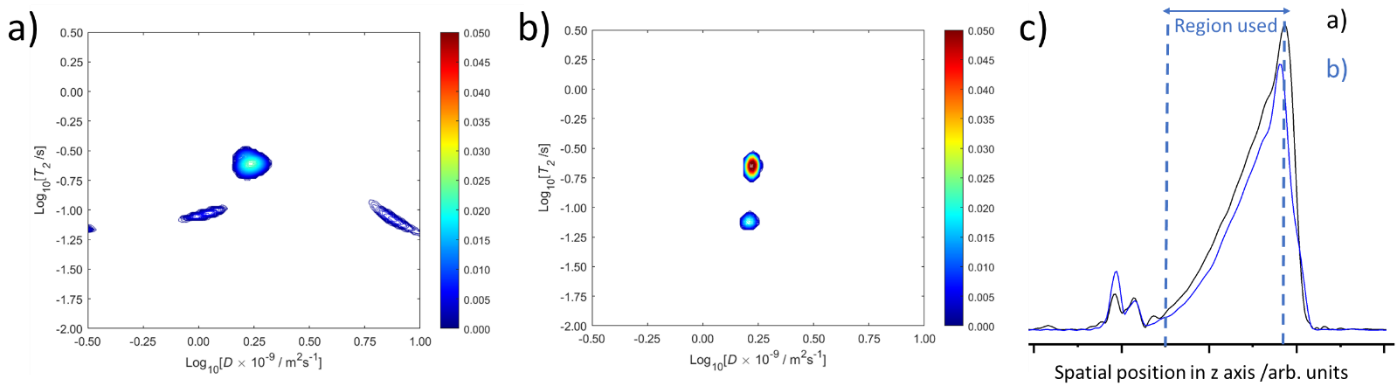

Figure S15: Comparison between repeated SABRE hyperpolarised *D*-T_2 correlation plots (CPMG loop) and spectral excitation detection profiles for two separate solutions of [IrCl(COD)(IMes)] (5 mM) and pyridine (10 equiv.) in methanol-*d_4* (0.6 mL) shaken with pH_2 (3 bar) for 10 seconds at 65 G without a delay between sample insertion and spectral acquisition. c) overlaid spectral excitation detection profiles.

S6. References

[1] L. A. Stables, R. P. Kennan, A. W. Anderson, J. C. Gore, *J. Magn. Reson.* **1999**, *140*, 305–314.

[2] R. W. Adams, J. A. Aguilar, K. D. Atkinson, M. J. Cowley, P. I. P. Elliott, S. B. Duckett, G. G. R. Green, I. G. Khazal, J. López-Serrano, D. C. Williamson, *Science* **2009**, *323*, 1708–1711.

[3] K. M. Appleby, R. E. Mewis, A. M. Olaru, G. G. R. Green, I. J. S. Fairlamb, S. B. Duckett, *Chem. Sci.* **2015**, *6*, 3981–3993.

[4] L. S. Lloyd, A. Asghar, M. J. Burns, A. Charlton, S. Coombes, M. J. Cowley, G. J. Dear, S. B. Duckett, G. R. Genov, G. G. R. Green, *Cat. Sci. Technol.* **2014**, *4*, 3544–3554.

[5] V. Daniele, F. Legrand, P. Berthault, J. Dumez, G. Huber, *ChemPhysChem* **2015**, *16*, 3413–3417.

[6] M. A. Javed, S. Ahola, P. Håkansson, O. Mankinen, M. K. Aslam, A. Filippov, F. U. Shah, S. Glavatskih, O. N. Antzutkin, V.-V. Telkki, *Chem. Commun.* **2017**, *53*, 11056–11059.

[7] S. Ahola, O. Mankinen, V.-V. Telkki, *Magn. Reson. Chem.* **2017**, *55*, 341–347.

[8] I. J. Day, *J. Magn. Reson.* **2011**, *211*, 178–185.

[9] P. M. Richardson, S. Jackson, A. Parrott, A. Nordon, S. B. Duckett, M. E. Halse, *Magn. Reson. Chem.* **2017**, *56*, 641–650.

[10] R. E. Mewis, K. D. Atkinson, M. J. Cowley, S. B. Duckett, G. G. R. Green, R. A. Green, L. A. R. Highton, D. Kilgour, L. Lloyd, J. A. B. Lohman, D. C. Williamson, *Magn. Reson. Chem.* **2014**, *52*, 358–369.

[11] P. Štěpánek, C. Sanchez-Perez, V.-V. Telkki, V. V Zhivonitko, A. M. Kantola, *J. Magn. Reson.* **2019**, *300*, 8–17.

[12] J. Hövener, S. Bär, J. Leupold, K. Jenne, D. Leibfritz, J. Hennig, S. B. Duckett, D. von Elverfeldt, *NMR Biomed.* **2013**, *26*, 124–131.
